# Antinociceptive effect of extracts of *Marrubium astracanicum* Jacq. aerial parts

**Published:** 2017

**Authors:** Niloofar Kahkeshani, Milad Gharedaghi, Abbas Hadjiakhoondi, Mohammad Sharifzadeh, Mahnaz Khanavi

**Affiliations:** 1 *Department of Pharmacognosy, Faculty of Pharmacy and Persian Medicine and Pharmacy Research center, Tehran University of Medical Sciences, Tehran, Iran*; 2 *Departments of Pharmacognosy and Medicinal Plants Research Center, Faculty of Pharmacy, Tehran University of Medical Sciences, Tehran, Iran*; 3 *Departments of Pharmacology and Toxicology and Pharmaceutical Sciences Research Center, Faculty of Pharmacy, Tehran University of Medical Sciences, Tehran, Iran*; 4 *Faculty of Land and Food Systems, University of British Columbia, B.C., Canada*

**Keywords:** Marrubium astracanicum, Pain measurement, Analgesic

## Abstract

**Objective:**

The genus *Marrubium* is used for treatment of joint pain, gout, stomach-ache and colic in Iranian Traditional Medicine. *Marrubium astracanicum* Jacq. (*M. astracanicum*) is a native species in the flora of Iran. The aim of this study was to evaluate the antinociceptive properties of various extracts of aerial parts of *M. astracanicum*.

**Materials and Methods:**

Antinociceptive activities of total hydroalcoholic extract (THE) and its n-hexane (non-polar) and residual partition (polar) fractions were analyzed using formalin test in mice. Morphine (5 mg/kg) and normal saline were used as positive and negative controls, respectively.

**Results:**

Intraperitoneal administration of THE (50, 100 and 200 mg/kg), non-polar fraction (200 mg/kg) and polar fraction (100 and 200 mg/kg), 30 min before formalin injection, caused significant analgesic activity in acute phase (0-5 min after formalin injection) of formalin test (p<0.05 as compared to control and p>0.05 in comparison with morphine). In chronic phase (15–60 min after formalin injection), non-polar and polar fractions (50, 100 and 200 mg/kg) showed significant analgesic activity (p<0.001 as compared to control and p>0.05 in comparison with morphine).

**Conclusion:**

Different extracts of *M. astracanicum* demonstrated antinociceptive activity that support the traditional usage of *Marrubium* genus for the treatment of arthritis, gout and other inflammatory diseases.

## Introduction

*Marrubium* L. (Lamiaceae) has about forty species, generally distributed in temperate regions of Asia, Europe and South America, of which eleven species are found in the flora of Iran. Generally, these plants are large annual or perennial shrubs, which are traditionally used to treat asthma, pulmonary infections, gastrointestinal disorders, insomnia and different types of pain and inflammation like joint pain and gout, in different countries (Jamzad, 2015 ▶; Mahdizadeh, 2015 ▶). 

Various studies showed several biological and pharmacological potencies such as antioxidant, anti-microbial, anti-hypertensive, anti-diabetic, analgesic and anti-inflammatory activities of different species of this genus. In most cases, these activities are related to valuable phytochemicals like flavonoids, phenylpropanoids, sterol and diterpenes, produced in *Marrubium* species (Meyre-Silva and Cechinel-Filho, 2010 ▶). 

*Marrubium astracanicum* Jacq. is a perennial shrub that grows in Turkey, Azerbaijan, Armenia, Georgia, Iraq and Iran. Decoction of its aerial parts is used to treat stomach-ache and joint pain in Iranian folk medicine (Jamzad, 2015 ▶; Mosaddegh et al, 2012 ▶). Methanol extract of *M. astracanicum* showed mild to moderate antioxidant potency, antibacterial activity against *Staphylococcus aureus*, *Sarcinalutea*, *Enterobacter aerogenes* and *Escherichia coli* and inhibitory activity against aldose reductase and platelet aggregation (Enomoto et al., 2004 ▶; Kunduhoglu et al., 2011 ▶; Tlili et al., 2013 ▶). Methanol and ethanol extracts of *M. astracanicum* showed an antitumor activity in *Agrobacterium tumefaciens*-induced potato disc tumor assay that was comparable to camptothecin as the reference standard (Yildirim et al, 2012 ▶). 

Phytochemical investigation of methanol extract of *M. astracanicum* resulted in isolation of two new labdane diterpenoids, Marrubinones A and B (Ilda et al., 1995 ▶). Three studies evaluated the essential oil constituents of this species in Iran and reported various profiles due to differences in inhabitance, collection time and climate. The major volatile components of sample, collected from Tehran province, Iran were caryophyllene oxide (35.8%), citronellal (16.9%) and β-caryophyllene (13.1%) while the essential oil of sample, gathered from Mazandaran province, Iran was mostly composed of methylcyclopentane (15.5%), thymol (10.6%) and n-heptane (7.4%) and the volatile oil of sample from Dena Mountain in the southwest of Iran, mainly consisted of β-caryophyllene (21.2%) and valeranone (5.4%) (Javidnia et al., 2007 ▶; Morteza-Semnani et al., 2004 ▶; Nik et al., 2003 ▶). 

The goal of this study was to assess the antinociceptive activity of total hydroalcoholic extract (THE) and n-hexane (non-polar) and residual (polar) partition fractions of *M. astracanicum* aerial parts to verify a traditional application of plants of *Marrubium* genus.

## Materials and Methods:

**Plant material**

Aerial parts of *M. astracanicum *were collected from Ardabil province, Iran in July 2010. A voucher specimen (IMPH-1443) was deposited in the Herbarium of Medicinal Plants Institute, Academic Centre for Education, Culture and Research (ACECR), Alborz, Iran. 

The air-dried and ground aerial parts (300 g) were extracted with methanol:water (80:20 % v/v, 3×1 l) at room temperature. The extract was concentrated using a rotary evaporator to give a dark green residue (30 g). A part of this extract (20 g) was suspended in the minimum volume of methanol 80% and partitioned by n-hexane which resulted in n-hexane (1 g) and residual (19 g) fractions after removal of solvent by a rotary evaporator. 

**Animals**

Male albino mice weighing 25-30 g were housed in groups of 6 with defined light-dark cycles (12 hr light and 12 hr dark), temperature (22±2 ºC) and access to food and water, *ad libitum*. They were allowed to accommodate with the laboratory condition 30 min before experiment start point. This study was ethically approved by the Ethics Committee of Pharmaceutical Science Research Centre, Tehran University of Medical Sciences (TUMS), Tehran, Iran.

**Administration of extracts in formalin test**

Groups of mice (n=6) were injected intraperitoneally with the extracts (50, 100 and 200 mg/kg; suspended or dissolved in normal saline), morphine 5 mg/kg (positive control) or normal saline (negative control). After 30 min, they were injected subcutaneously with 25 µl of formalin solution (2.5%) into their paws. Mice were observed in a cylindrical glass chamber of 20 cm diameter, with a mirror at a 45° angle for clear observation of their paws and the total time (sec) spent for licking the injected paw in the acute (0-5 min) and chronic (15-60 min) phases was calculated as a pain indicator. Pain inhibition percentage (%) was calculated by the following expression:

$\frac{\left(\mathrm{mean of control group})–(\mathrm{mean of treated group}\right)}{\left(\mathrm{mean of control group}\right)}\times 100$

***Statistical analysis***

Results were expressed as mean±S.D. Statistical difference among groups was evaluated using one-way ANOVA followed by Tukey´s post hoc. A p value less than 0.05 was considered statistically significant. 

## Results

**Figure 1:**
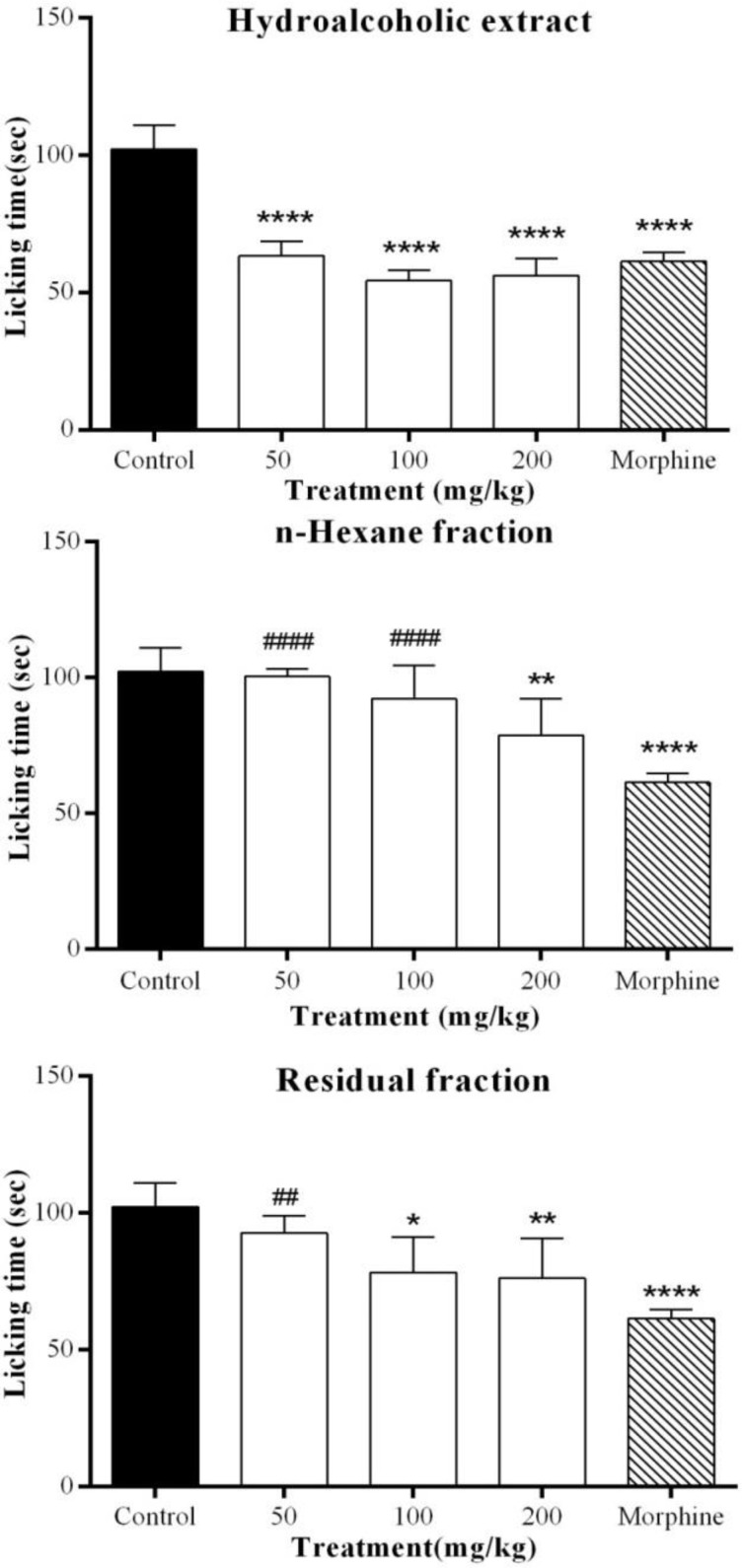
Effect of different extracts of *Marrubium astracanicum* aerial parts on acute phase of formalin-induced pain. Different doses of all extracts were administered intraperitoneally to mice, 30 min before intraplantar injection of formalin. Antinociception was recorded 0-5 min after formalin injection. Control group received normal saline. Values are mean±S.D. for each group of six mice. *p<0.05, **p<0.01, ***p<0.001, ****p<0.0001 (as compared to control), # p<0.05, ##p<0.01, ###p<0.001 and #### p<0.0001 (as compared to morphine 5 mg/kg

**Figure 2:**
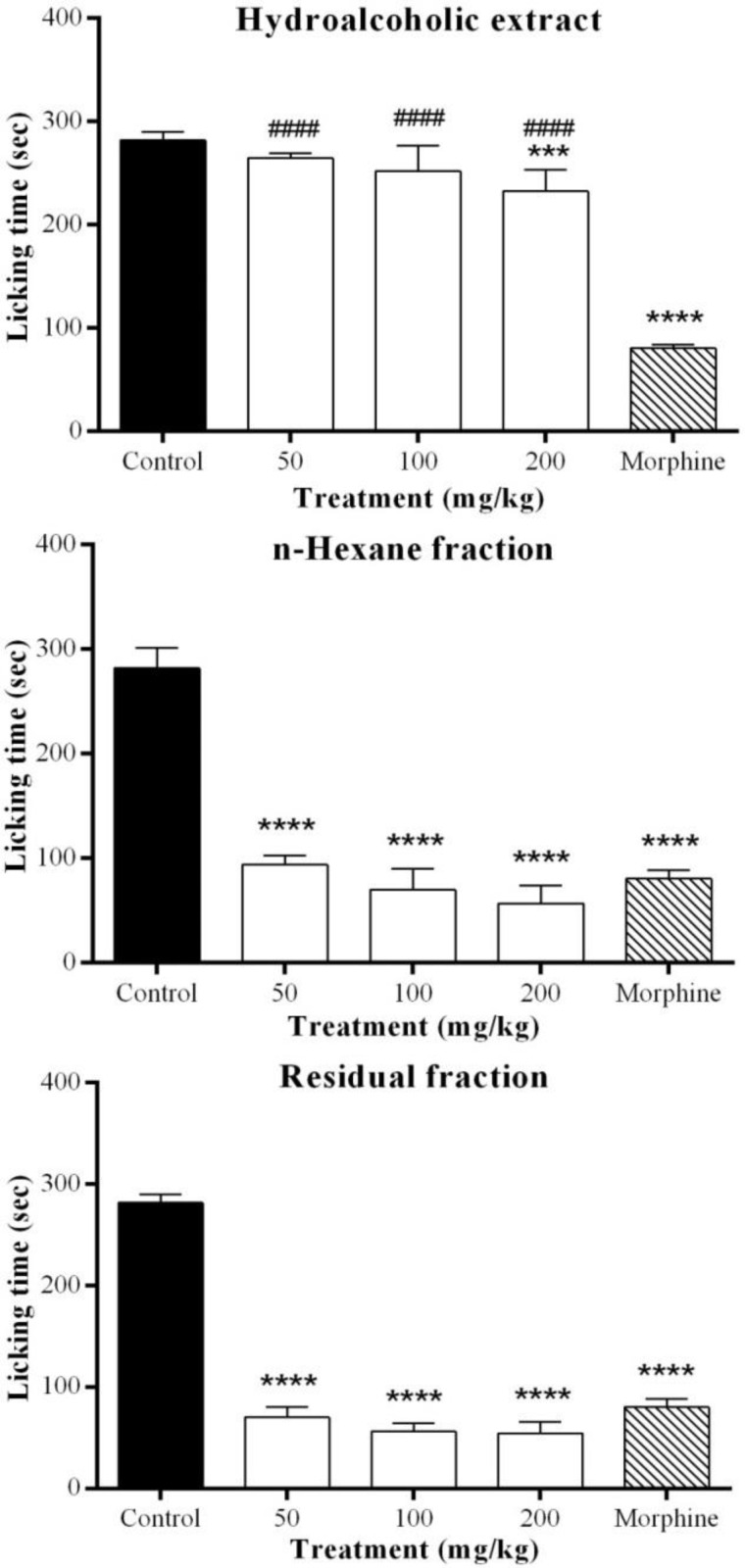
Effect of different extracts of *Marrubium astracanicum* aerial parts on chronic phase of formalin-induced pain. Different doses of all extracts were administered intraperitoneally to mice, 30 min before intraplantar injection of formalin. Antinociception was recorded 15-60 min after formalin injection. Control group received normal saline. Values are mean±S.D. for each group of six mice. *p<0.05, **p<0.01, ***p< 0.001, ****p<0.0001 (as compared to control), # p<0.05, ##p<0.01, ###p<0.001 and #### p<0.0001 (as compared to morphine 5 mg/kg

In this study, analgesic activity of *Marrubium astracanicum* aerial parts hydroalcoholic extract and its main fractions was assessed in a pain model in mice. All extracts were administered intraperitoneally at three doses (50, 100 and 200 mg/kg) 30 min before subcutaneous intraplantar injection of formalin (25 µl, 2.5%). Figures 1 and 2 show the licking time in acute and chronic phases of formalin test, respectively. THE (50, 100 and 200 mg/kg) and polar fraction (100 and 200 mg/kg) significantly decreased licking time (p˂0.05) in acute phase and their activity was comparable to morphine (5 mg/kg, p>0.05). Similarly, non-polar fraction showed comparable analgesic activity to morphine in acute phase at the dose of 200 mg/kg (p˂0.01 as compared to control and p>0.05 in comparison with morphine).

In chronic phase, THE (200 mg/kg) significantly decreased licking time (p˂0.001) but its analgesic effect was not comparable to morphine 5 mg/kg ( p<0.0001). Non-polar fraction (50, 100 and 200 mg/kg) showed an analgesic activity in this phase (p<0.0001) which was comparable to morphine 5 mg/kg (p>0.05). Similarly, polar fraction (50, 100 and 200 mg/kg) exhibited a significant reduction on the duration of licking time in chronic phase (p<0.0001) which was comparable to morphine 5mg/kg (p>0.05).

Table 1 shows inhibition percentage of all extracts at different doses. THE was significantly more effective in inhibition of pain in early phase (p<0.001), while non-polar and polar fractions were significantly more active in late phase (p<0.0001).

**Table 1:** Inhibition percentage of different extracts of *Marrubium astracanicum* aerial parts in formalin test

**Groups (n=6)**	**Dose (mg/kg)**	**Inhibition percentage (%)**	
		**Acute phase**	**Chronic phase**
**Total extract**	50	38.03±5.2 ****	6.21±4.29 ####
	100	46.83±3.59 ***	10.83±8.85 ####
	200	45.03±6.17 ****	17.58±7.55 ####
**n-Hexane fraction**	50	1.85±6.71 ####	66.78±3.05 ****
	100	9.98±12.07 ###	75.37±7.26 ****
	200	23.02±13.10	79.92±6.12 ****
**Residual fraction**	50	9.4±15.2 ###	75.07±8.67 ****
	100	23.5±12.65	80.10±9.11 ****
	200	25.63±14.22	80.63±4.02 ****
**Morphine **	5	39.99±3.25	71.58±2.98

Different doses of all extracts were administered intraperitoneally to mice, 30 min before intraplantar injection of formalin. Antinociception was recorded 0-5 min (acute phase) and 15-60 min (chronic phase) after formalin injection. Values are mean±S.D. for each group of six mice.

* p<0.05,

** p<0.01,

*** p<0.001,

**** p<0.0001 (as compared to acute or chronic phase),

# p<0.05,

## p<0.01,

### p<0.001

#### p<0.0001 (as compared to morphine 5 mg/kg).

## Discussion

Formalin test is an appropriate model for simulation of pain in clinical conditions. In this test, subcutaneous injection of formalin provides a mild to moderate and continuous pain due to tissue injury, which has two separate phases. Acute early phase, 0-5 min after formalin injection, is an indicator of centrally mediated pain related to direct stimulation of nociceptors and C fibers beside the effects of substance P and bradykinin while chronic late phase, 15-60 min after injection, is mainly due to secretion of inflammatory mediators like histamine, serotonin, bradykinin and prostaglandins plus sensitization of central pain neurons. Centrally acting analgesic like opioids control both phases equally. In contrast, peripherally acting drugs like corticosteroids and NSAIDs only inhibit the late phase (Tjolsen et al., 1992 ▶).

In a previous study, oral and intraperitoneal administration of *M. vulgare *hydroalcoholic extract (10, 30 and 60 mg/kg), showed desirable dose-dependent analgesic activity in writing test and late phase of formalin test in mice. Authors concluded that the extract worked through a non-opioid way by inhibiting prostaglandin synthesis. They attributed this effect to the presence of some phytochemicals like steroids and terpenes (de Suza et al., 1998 ▶). Later, they investigated the analgesic activity of marrubiin, a labdane diterpene which was isolated from *M. vulgare*. Marrubiin and its derivative, marrubiinic acid, showed significant analgesic activity in both writing and formalin tests. They were even more active than some clinically used drugs like aspirin at same doses. Authors could not clarify the precise mechanism of action but they concluded that it was unlikely to be associated with the opioid way. Finally, they proposed some undetermined central and peripheral pathways e.g. inhibition of excitatory amino acids like glutamate, inhibition of different pro-inflammatory or phlogistic agents like bradykinin, histamine and substance P and antagonistic effects on vanilloid pain receptors (de Jesus et al., 1999 ▶; Meyre-Silva et al., 2005 ▶). Moreover, different phenylpropanoids isolated, from methanolic extract of *M. vulgare* has shown potent analgesic activity via selective inhibition of COX-2 (Sahpaz et al., 2001 ▶). 

In another study, oral administration of *M. globosum* ssp. *libanoticum* acetone extract (10, 30 and 100 mg/kg), significantly inhibited carrageenan-induced rat paw edema by inhibiting COX-2 and iNOS enzymes activity. Bioassay-guided fractionation of this extract led to isolation of a bioactive labdane diterpene, marrulibanoside (Rigano et al., 2006 ▶). A recent study reported that hydroalcoholic extract of *M. parviflorum* at the dose of 200 mg/kg significantly inhibited the chronic phase of formalin test probably by its phytochemicals like terpenoids and phenolic compounds (Khanavi et al., 2012 ▶). 

Our results showed that total hydroalcoholic extract of *M. astracanicum* decreased the pain in both acute and chronic phases and its activity was more marked in acute phase but both non-polar and polar fractions were significantly more active in chronic phase. Total hydroalcoholic extract is a complex mixture of different phytochemicals with a wide range of polarity. Fractionation procedure with different solvents (from non-polar to polar) can partially separate the phytochemicals based on their relative polarity. So, non-polar phytochemicals like terpenoids are more concentrated in non-polar fraction while polar ones are mainly in polar fraction (Harborne AJ, 1998 ▶). According to this explanation and earlier studies, the significant analgesic activity of non-polar and polar fractions of *M. astracanicum* in chronic phase of formalin test can be partly attributed to inhibitory activity of their respective major phytochemicals *i.e*. terpenoids and phenolics on peripheral inflammation mediators like histamine, serotonin and prostaglandins. As mentioned previously, total hydroalcoholic extract of *M. astracanicum* was significantly more active in acute phase and it was almost inactive in chronic phase. Lack of analgesic activity in chronic phase can be related to competitive and antagonistic impact of different phytochemicals on inhibition of enzymes like COX-2 and i-NOS. 

In conclusion, both polar and non-polar fractions obtained from total hydroalcoholic extract of *M. astracanicum* aerial parts showed promising antinociceptive properties. This activity is probably mediated through inhibition of endogenous inflammatory mediators especially prostaglandins. According to earlier studies, major phytochemicals of this genus (*i.e.* steroids, labdane diterpenes and phenolics) can be responsible for antinociceptive effect. Moreover, this study can confirm the traditional use of some *Marrubium* species for treatment of joint pain, gout and other inflammatory problems.
